# Industrial Winemaking Waste to Sustainable Palladium(II) Recovery: A Green One-Step Synthesis of Activated Carbon from Grape Seeds

**DOI:** 10.3390/ma19010107

**Published:** 2025-12-28

**Authors:** Tomasz Michałek, Maciej Mańka, Marek Wojnicki

**Affiliations:** 1 Faculty of Non-Ferrous Metals, AGH University of Krakow, al. A. Mickiewicza 30, 30-059 Cracow, Poland; tomaszm@agh.edu.pl; 2 Mineral and Energy Economy Research Institute, Polish Academy of Sciences, 7A Wybickiego Street, 31-261 Cracow, Poland; mmanka@min-pan.krakow.pl

**Keywords:** palladium, adsorption, activated carbon, grape seeds, pyrolysis, sustainability

## Abstract

The growing demand for palladium (Pd) necessitates the development of sustainable and efficient recovery methods. This work presents a green, one-step synthesis of activated carbon (AC) from winemaking waste (grape seeds) via direct pyrolysis, eliminating the need for separate, energy-intensive activation. Remarkably, the AC synthesized at the lowest temperature of 400 °C exhibited the highest Pd(II) adsorption capacity (16.20 mg/g at 50 °C), performing comparably to many literature-reported ACs that underwent complex activation processes. Characterization revealed that this optimal material possessed a favorable point of zero charge (PZC 7.78) and the lowest ash content (4.66%). Higher pyrolysis temperatures (400–800 °C) progressively increased surface basicity (PZC up to 11.00) and carboxylic group content (reaching 0.565 mmol/g at 800 °C). A comprehensive life cycle assessment (LCA) demonstrated the significant environmental advantage of this method, showing a 74% lower total environmental impact and a 92% reduction in acidification potential compared to commercial coal-based AC. These results prove that highly effective Pd(II) recovery can be achieved through a simplified, direct pyrolysis process, offering a sustainable and practical approach for precious metal recycling from waste biomass.

## 1. Introduction

Palladium (Pd) is a platinum-group metal (PGM) known for its unique physical and chemical properties, playing a critical role in industries such as automotive catalysis [1], electronics [2], and chemical synthesis [3]. Its economic importance and limited natural reserves make efficient recovery and recycling essential [4]. Moreover, the contribution of all PGMs to the advancement of the global economy is expected to drive a continued rise in their consumption, further intensifying the demand for these metals [5]. Given its indispensable role in the clean energy transition, particularly in hydrogen technologies, securing a sustainable supply of Pd is a growing global imperative [6]. A common challenge in Pd recovery is its extraction from diluted solutions, such as industrial wastewater or leachates from spent catalysts, where traditional methods like precipitation or solvent extraction often prove inefficient, expensive, or environmentally harmful due to the use of toxic chemicals and generation of secondary waste [7,8]. Adsorption has gained attention as a practical and scalable alternative for recovering metal ions from such solutions [9,10].

Activated carbon (AC) is a widely used adsorbent for metal recovery due to its large surface area, porous structure, and adaptable surface chemistry [11]. While AC is traditionally produced from fossil-based materials [12], it can also be made from renewable biomass sources, offering a more sustainable option [13]. Agricultural and food waste, such as coconut shells [14], cherry seeds [15], and rice husks [16], have been widely explored as precursors for AC production. The winemaking process—including grape pressing and maceration—yields not only wine but also a significant amount of organic waste. This waste primarily consists of grape seeds, followed by lees, stems, and dehydrated sludge [17]. Grape seeds are particularly promising candidates for AC production due to their abundance and suitability for creating effective adsorbent materials [18]. The utilization of this abundant, low-cost agro-industrial residue aligns perfectly with the principles of the circular economy and waste valorization [19]. Previous research has demonstrated the versatility of grape seed-derived AC for the adsorption of various heavy metal ions, such as Pb [20], Cd [21], and Cu [22]. While previous studies have utilized grape seeds for AC production, they often rely on a separate activation process to enhance the material’s properties [23,24,25,26]. In contrast, this study introduces a one-step synthesis approach, demonstrating that biomass pyrolysis alone can produce AC with effective adsorption properties, eliminating the need for additional activation steps. Conventional activation methods, such as chemical treatment with strong acids or bases or physical activation using high-temperature steam or CO_2_, often require significant energy input or hazardous chemicals [27], which conflict with green chemistry principles [28]. By avoiding these steps, the one-step pyrolysis method not only simplifies the production process but also reduces environmental impact, offering a more sustainable alternative for AC synthesis.

In this study, we investigate the one-step synthesis of AC from grape seeds and assess its effectiveness in adsorbing Pd(II) ions from aqueous solutions. The synthesized AC is thoroughly analyzed, with a focus on its structural, morphological, and surface properties, as well as the environmental impact associated with the production of this material. This work aims to advance the development of sustainable and efficient materials for precious metal recovery by focusing on a detailed thermodynamic analysis of Pd(II) adsorption. Thermodynamic parameters provide fundamental insight into adsorption equilibrium, the endothermic or exothermic nature of the process, and the feasibility and stability of metal–adsorbent interactions, which are critical for evaluating the real-world applicability of the synthesized materials in Pd(II) recycling.

## 2. Materials and Methods

### 2.1. Activated Carbon Synthesis

Grape seeds were initially crushed and subsequently dried at 100 °C for 6 h using a drying oven (VWR Collection, Lutterworth, UK). The seeds were then pulverized for 1 min in a laboratory roller-ring mill (EKO–LAB, Brzesko, Poland). Following this, the ground seeds were roasted in a tube furnace (Czylok, Jastrzębie-Zdrój, Poland) under a N_2_ atmosphere (99.999% purity, Air Liquide, Kraków, Poland), with a gas flow rate of 20 L/h. To ensure an inert atmosphere, a 30 min waiting period was maintained before initiating the heating process to rinse the furnace chamber. The time to reach the set temperature was constant (1 h), after which the samples were held at the target temperature for 3 h. Following pyrolysis, the samples were cooled to room temperature in the furnace under continuous N_2_ flow. Samples 1–5 were synthesized at temperatures of 400, 500, 600, 700, and 800 °C, respectively. To thoroughly evaluate the characteristics and performance of the AC, a series of analytical techniques was utilized.

### 2.2. Ash Content and Composition

Ash content analysis was conducted for each sample by heating in the same tube furnace used for synthesis, but under an air atmosphere. The temperature was gradually increased to 800 °C over 12 h, achieving an approximate heating rate of 1 °C per minute. This controlled heating process prevented graphitization of the samples. The ash content was then determined using Equation (1):

(1) $$Ash\;content\;[\%]=\frac{m_{ash}}{m_{carbon}}\times 100\%$$

In addition to determining the ash content percentage in the samples, the resulting ash was further analyzed using Energy Dispersive X-Ray Fluorescence (EDXRF) spectroscopy (MINIPAL 4, PANalytical B.V., Espoo, Finland) to evaluate its chemical composition.

### 2.3. Morphology and Surface Area

The microstructure and elemental composition of the AC samples were analyzed using a JEOL-6000 Plus instrument (Tokyo, Japan) operated at an accelerating voltage of 15 kV. For each sample, a single small piece of AC was crushed to expose both the outer and inner structures, ensuring a representative analysis of the material’s morphology. The prepared samples were mounted on a sample holder and analyzed under high vacuum. Images were captured at two magnifications (×500 and ×2000) to examine both the overall morphology and fine structural details. Energy-dispersive X-ray spectroscopy (EDS) was performed on selected regions to determine the elemental composition.

BET surface area analysis was performed using ASAP 2010 instrument (Micromeritics Instrument Corporation, Norcross, GA, USA). The samples were degassed at 200 °C under a vacuum of 4 µmHg for 24 h to remove adsorbed impurities. Measurements commenced after achieving a vacuum of 10 µmHg in the analysis cell, with the initial data point recorded at a pressure of 40–50 mmHg. The analysis was conducted at a constant temperature of 77.35 K.

### 2.4. X-Ray Diffraction

The phase composition of the ACs was characterized by X-ray diffraction (XRD). Measurements were performed using a Rigaku MiniFlex II diffractometer (Rigaku Corporation, Tokyo, Japan) equipped with a Cu Kα radiation source (λ = 1.54059 Å). Patterns were recorded in the 2θ range of 10° to 60° with a step size of 0.02° and a scanning speed of 0.5°/min.

### 2.5. Functional Groups

Fourier-transform infrared (FT-IR) spectroscopy was carried out using a Nicolet 380 spectrometer (Waltham, MA, USA). The transmission method was employed for spectral analysis. Samples were prepared by grinding 0.2 g of KBr (Merck, Darmstadt, Germany) with a very small amount of AC in an agate mortar. Although precise mass measurements of the AC samples were not required for this qualitative analysis, the consistent mass of KBr ensured uniformity in pellet preparation. Pellets were formed using a 10 mm diameter punch and a hydraulic press applying a pressure of up to 10 MPa.

The surface chemistry of the AC samples was further characterized using Boehm titration to quantify the concentration of acidic and basic functional groups. Solutions of NaHCO_3_ (0.1 M), Na_2_CO_3_ (0.05 M), NaOH (0.1 M), C_2_H_5_ONa (0.1 M), and HCl (0.1 M) were prepared and standardized prior to the analysis. For every sample, 0.5 g of AC was added to 100 mL of each solution and shaken for 24 h at room temperature using a shaker (ELPIN, Lubawa, Poland). After filtration, 25 mL of the filtrate was taken twice for analysis. The remaining base or acid in the solution was titrated conductometrically using a glass burette to determine the amount of functional groups present. The results were averaged to obtain the final concentrations of acidic (carboxylic, lactonic, phenolic, and carbonyl) and basic functional groups.

### 2.6. Point of Zero Charge

To determine the point of zero charge (PZC) for the ACs produced under varying synthesis parameters (as outlined in Section 2.1), solutions with pH values ranging from 2 to 12 were prepared. These solutions were formulated using 0.1 M NaNO_2_, adjusted to the desired pH levels with 0.1 M HClO_4_ for acidification and 0.1 M NaOH for alkalization. The pH measurements during PZC determination were conducted using a multifunctional ELMETRON CX–505 m (Zabrze, Poland). Prior to measurements, the device was calibrated using the three-point method with standard buffer solutions at pH 1, 4, and 7 (POCH S.A., Gliwice, Poland).

### 2.7. Pd(II) Adsorption

A stock solution containing Pd(II) ions (Pd concentration: 0.112714 M) was prepared using a previously reported procedure [29]. Further dilutions of the stock solution were made using 0.1 M HCl to obtain Pd(II) concentrations of approximately 0.1, 0.2, 0.5, and 1 mM (detailed concentrations are present in Supplementary Data, Table S1). Given the presence of Cl^−^ anions, Pd(II) ions were present as coordination complexes in the system [30]. These solutions were used to conduct adsorption tests on the AC derived from grape seeds. For each measurement series, approximately 0.334 g of AC was added to 200 mL of the Pd(II) solution. The mixtures were shaken for 3 days at controlled temperatures (30, 40, and 50 °C) using a shaker (ELPIN, Lubawa, Poland). The equilibrium concentrations of Pd(II) at each temperature were analyzed using a Jasco V-770 spectrophotometer (Tokyo, Japan).

The amount of Pd(II) adsorbed per unit mass of AC, referred to as the adsorption load, was calculated using Equation (2):

(2) $$q_{e}=\frac{(C_{0}-C_{e})V}{m}$$

where *q_e_*—adsorption load at equilibrium [mg/g], *C*_0_—initial Pd(II) concentration [mg/L], *C_e_*—equilibrium Pd(II) concentration [mg/L], *V*—solution volume [L], and *m*—AC mass [g].

The adsorption data were analyzed using the Freundlich and Temkin isotherm models. The Freundlich isotherm is widely used to describe adsorption on heterogeneous surfaces, as it accounts for the non-uniform distribution of adsorption sites and varying adsorption energies [31]. The model is based on the empirical equation proposed by Freundlich:

(3) $$q_{e}=K_{F}C_{e}^{\frac{1}{n}}$$

where *K_F_*—Freundlich adsorption constant [(mg/g)(L/mg)1/n], and *n*—adsorption intensity (dimensionless).

Equation (3) can be rearranged into linear form, enabling the calculation of isotherm parameters from the slope and intercept of linear regression. The linearized form of the Freundlich isotherm is presented in Equation (4):

(4) $$\log q_{e}=\log K_{F}+\frac{1}{n}\log C_{e}$$

The Temkin isotherm incorporates a factor that explicitly accounts for adsorbent–adsorbate interactions. This model assumes that the heat of adsorption of all molecules in the layer decreases linearly with coverage, rather than logarithmically [32]. This isotherm is described by the following equation:

(5) $$\log q_{e}=\frac{RT}{b_{T}}\ln A_{T}+\frac{RT}{b_{T}}\ln C_{e}$$

where *R*—universal gas constant [J/mol·K], *T*—temperature [K], *b_T_*—Temkin constant [J/mol], and *A_T_*—Temkin isotherm constant [L/g].

### 2.8. Life Cycle Assessment

The comparative life cycle assessment (LCA) analysis was conducted using the SimaPro software (9.6.2), utilizing the Ecoinvent v3.10 database. All calculations were performed in accordance with the environmental footprint (EF) methodology. The electricity consumption was recalculated based on the specified heating capacity of the oven and the amount of thermal energy absorbed by the grape seeds during processing. The results are expressed per 1 kg of the final product. The entire assessment encompasses sixteen impact categories as defined by the EF method.

## 3. Results and Discussion

### 3.1. Ash Content and Composition Analysis

Ash content plays a crucial role in determining the quality of AC. An excessive amount of ash generally has a negative impact on its porosity [33], as ash is inherently non-porous and can obstruct or reduce the availability of pores [34]. However, on the other hand, a high ash content may indicate a significant inorganic fraction, which could function as a natural doping agent by introducing metals or minerals into the carbonaceous matrix. Such inorganic species might enhance catalytic properties in specific applications [35]. Nevertheless, for most adsorption-focused uses, a lower ash content in AC results in better performance. The results for the obtained AC samples are presented in Table 1.

AC samples produced from grape seeds are characterized by a relatively low ash content, averaging 5.37%, while the ash content in AC generally ranges from 1 to 20%, depending on the biomass used in the synthesis [36]. In the case of our samples, higher synthesis temperatures lead to an increase in ash content. The lowest ash content was obtained at 400 °C, as the milder pyrolysis at this temperature results in a higher biochar yield, effectively diluting the concentration of inorganic ash precursors within the solid [37]. Consequently, the resulting biochar has a lower relative concentration of non-volatile inorganic minerals (ash precursors) compared to biochars produced at higher temperatures, where more extensive volatilization leads to a greater concentration of these inorganics in the remaining solid [38]. However, this correlation is not strictly linear, as a peak ash content was observed at 500 °C, followed by slightly lower but comparable values at higher synthesis temperatures. Based on the ash contents measured at ≥500 °C (5.80, 5.34, 5.39, and 5.68%), the low standard deviation (≈0.22%) indicates that the differences in ash content above 500 °C are negligible and likely within experimental error, implying that biochar yield has largely stabilized beyond 500 °C and that further increases in synthesis temperature do not lead to additional concentration of the mineral fraction, whereas a noticeable change in ash content is only observed below 500 °C. The elemental composition of ashes was analyzed using the EDXRF method, and the results are presented in Table 2.

The EDXRF results for the ashes obtained from the AC samples show a diverse elemental composition, with Ca and P being the most prominent elements. These elements, along with K, Mg, and S, are commonly found in all living organisms, including plants [39], which explains their presence in the samples derived from grape seeds. Other trace elements such as Fe, Cu, Zn, Mn, Cr, and Ti are also detected, but at much lower concentrations. The significant P content across all temperatures, including the highest value at 400 °C (23.90%), indicates that the inherent P from the biomass is largely retained even at lower pyrolysis temperatures. This differs from the mechanism described for H_3_PO_4_-ACs, where P retention increases markedly above 500 °C [40]. In the present system, the peak in total ash content at 500 °C cannot be attributed solely to P retention, but rather to the combined concentration effect of all mineral constituents, with P serving as a major, stable component throughout the temperature range. Overall, the concentrations of both major and trace elements remain fairly consistent across the samples prepared at different pyrolysis temperatures, suggesting that the elemental composition of the ashes is largely preserved during AC production.

### 3.2. Morphology and Surface Area Analysis

**Figure 1:**
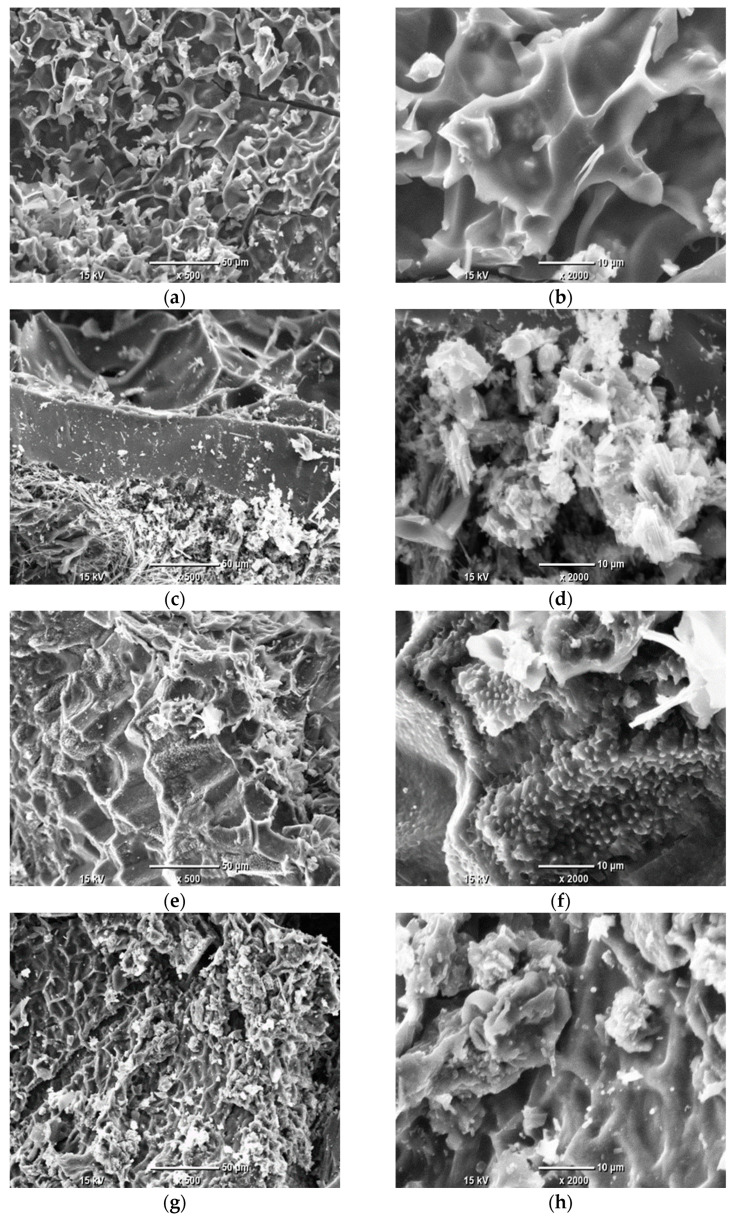

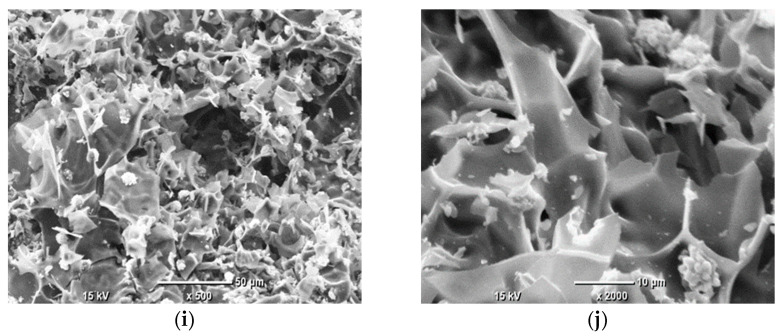
SEM images of AC samples under ×500 and ×2000 magnifications: Sample 1 (**a**,**b**); Sample 2 (**c**,**d**); Sample 3 (**e**,**f**); Sample 4 (**g**,**h**); Sample 5 (**i**,**j**).

The microstructure and elemental composition of the AC derived from grape seeds were analyzed using SEM and EDS. SEM imaging revealed the surface morphology, including features such as porosity, cracks, and particle structure, which are critical for understanding the material’s textural properties. Given that the AC is produced through the pyrolysis of grape seeds, it may potentially retain some structural features inherited from the original biomass. EDS provided complementary information on the elemental distribution, highlighting the presence of inorganic residues or heteroatoms that may influence the material’s performance. Together, these techniques offer a detailed view of the microstructural characteristics. The SEM images, presented in Figure 1, illustrate the morphological changes induced by pyrolysis temperature.

The SEM analysis revealed that the microstructure of the ACs derived from grape seeds is highly heterogeneous, exhibiting a diverse range of morphological features. In some regions, the surface displays acicular or fibrous formations (Figure 1c,d) likely remnants of the lignocellulosic framework of the original biomass, preserved during pyrolysis. Other areas show a lamellar or layered microstructure, characterized by overlapping plate-like features (Figure 1e,f). Across all samples, crystalline deposits are visible, likely corresponding to inorganic residues or impurities. Notably, no significant correlation was observed between the microstructure and the pyrolysis temperature, indicating that the morphological diversity is primarily influenced by the inherent structural properties of the grape seed precursor rather than the synthesis conditions.

**Figure 2:**
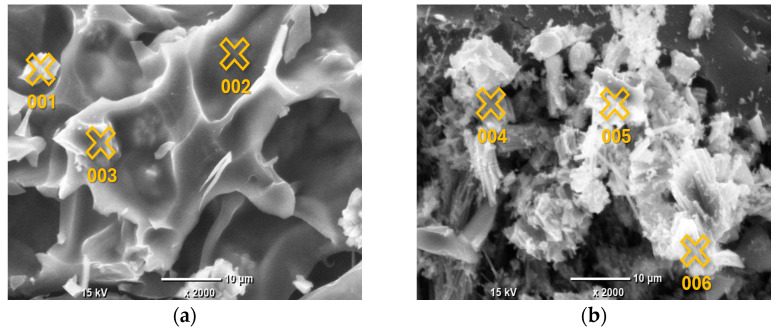

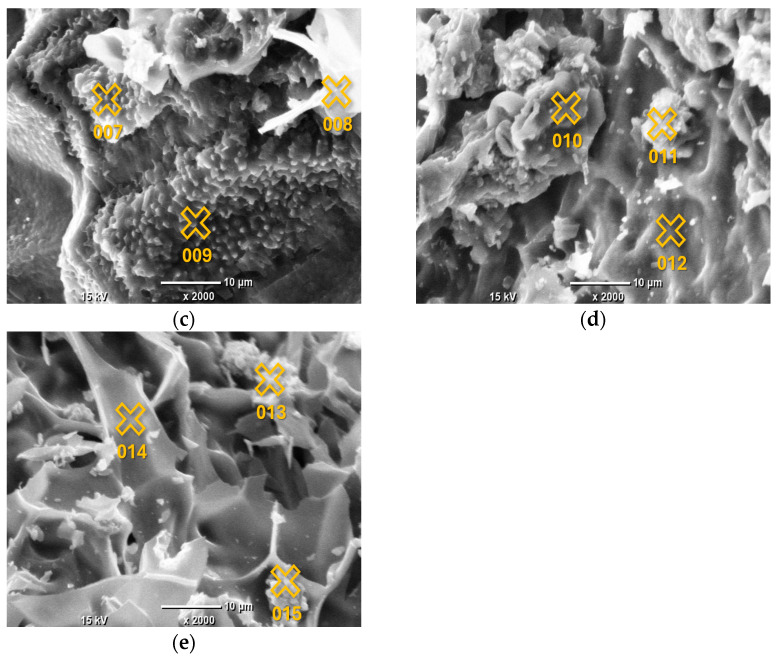
SEM images of AC samples under ×2000 magnification, with marked points of EDS analysis: Sample 1 (**a**); Sample 2 (**b**); Sample 3 (**c**); Sample 4 (**d**); Sample 5 (**e**). The marked points correspond to the locations listed in Table 3 for EDS analysis.

The EDS analysis focused on the elemental composition of the crystalline deposits observed in the SEM images, which appeared as white crystals across all AC samples. Given that the EDS results for these crystals were highly consistent across all samples, indicating a similar composition regardless of the pyrolysis conditions, the analysis of Sample 1 is provided as a representative example. The detailed results, including the identified elements and their relative concentrations, are presented in Figure 2 and Table 3.

The surface elemental composition of the grape seed-derived AC, as determined by EDS spot analysis, reveals a complex picture that is not defined by a simple linear trend of increasing C content with pyrolysis temperature. Instead, the data highlight two dominant and concurrent phenomena: the heterogeneous deposition of inorganic phases and the variable retention of heteroatoms within the carbonaceous matrix.

The most striking feature of the EDS data is the extreme heterogeneity in surface composition, evident across all synthesis temperatures. This is characterized by the presence of discrete points with C content below 60 at.% alongside points with C content exceeding 80 at.%. Points such as 011 (700 °C; 38.78 at.% C, 45.47 at.% Ca) and 004 (500 °C; 54.76 at.% C, 41.05 at.% O, 3.95 at.% Ca) are clearly not representative of the carbonaceous bulk but are localized inorganic or O-rich deposits. The composition of these deposits is highly consistent with the bulk ash analysis obtained by EDXRF (Table 2), which identified Ca, P, and K as the dominant inorganic constituents. For instance, the high-Ca signature of Point 011 directly corresponds to the major Ca component (59.58%) of the bulk ash. Similarly, Points 010, 012, and 015, which show notable levels of K and/or P alongside Ca, reflect the surface segregation of the other major ash components (P: 22.02%, K: 8.78%). This correlation confirms that the inorganic matter from the biomass precursor coalesces into distinct, heterogeneously distributed mineral phases on the surface during pyrolysis, rather than forming a homogeneous dispersion.

Regarding the carbonaceous phase, the EDS data do not show a systematic increase in surface C content with increasing pyrolysis temperature from 400 °C to 800 °C. For example, some of the highest C contents (>84 at.%) are recorded at 400 °C (Point 002) and 600 °C (Point 009), while some of the lowest (<66 at.%) are found at 700 °C and 800 °C (Points 010, 011, 015). This apparent lack of correlation suggests that at the microscale, the local surface chemistry is more strongly influenced by the proximity to inorganic deposits and the resulting contrast in atomic number, rather than by the average bulk carbonization degree.

**Figure 3:**
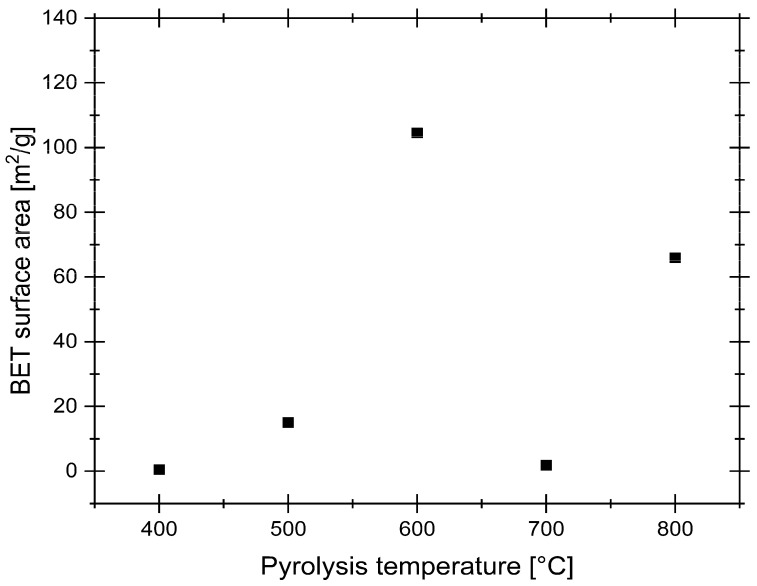
BET analysis results.

BET analysis is a crucial method for determining the surface area of AC, a key parameter that directly influences its adsorption capacity and overall performance in various applications. While a higher BET surface area generally indicates greater potential for physisorption, its direct translation to performance in aqueous metal ion recovery can be complex, as adsorption is also governed by surface chemistry and pore accessibility for hydrated species. Figure 3 presents the results obtained from the BET analysis.

The BET surface area analysis reveals a strong dependence on synthesis temperature. At lower temperatures, surface area remains minimal, suggesting incomplete pore development. As the temperature increases, a notable rise in surface area occurs, peaking at 600 °C, before declining again at higher temperatures due to potential pore collapse or excessive burn-off. However, the partial recovery observed at 800 °C indicates a more nuanced process than simple pore collapse. This trend likely reflects a dynamic competition in the 600–800 °C range between pore creation, pore widening, and structural reordering, where high temperatures may also remove amorphous C deposits, which create new mesopores [40]. Compared to commercial ACs, which typically exhibit surface areas in the range of 500–1500 m^2^/g, the obtained values are considerably lower. However, it is important to note that BET analysis reflects the ability of N_2_ to adsorb onto the sample surface and may not necessarily correspond to the material’s effectiveness in aqueous environments. Despite its low measured surface area, the samples performed adequately in subsequent adsorption studies for target metal ions. This suggests the adsorption process was governed not by total micropore volume, but by specific surface chemistry more favorable to Pd complexes.

### 3.3. XRD Analysis

**Figure 4:**
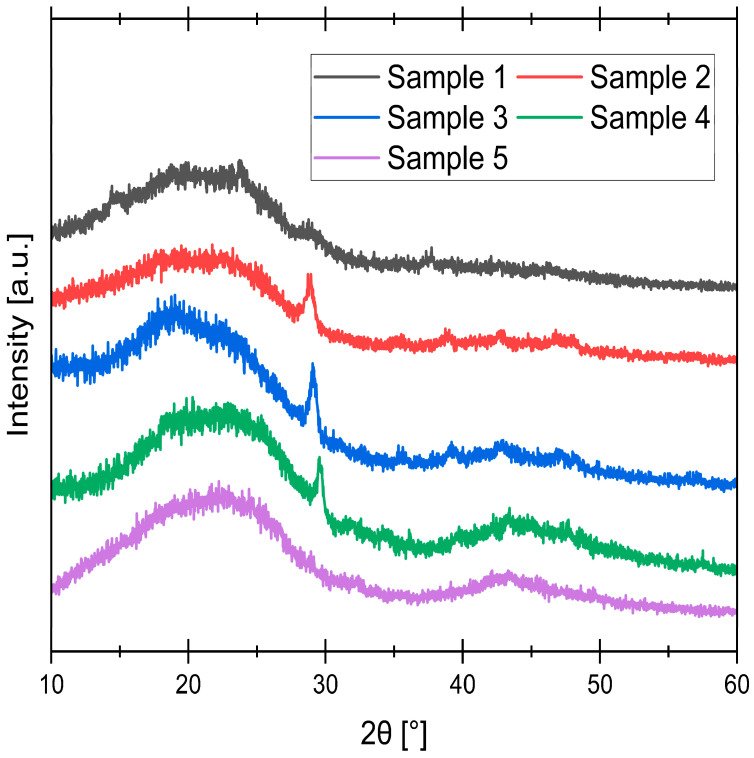
XRD spectra of synthesized samples.

XRD analysis provides essential insight into the crystallographic structure and phase composition of the synthesized samples. For ACs, XRD is a critical tool for assessing the degree of graphitic order and identifying inorganic crystalline impurities. The technique primarily reveals the long-range atomic arrangement, distinguishing between amorphous domains, disordered graphitic structures, and crystalline graphitic phases. Results are shown in Figure 4.

The persistent, broad diffraction peak near 22° observed across all samples corresponds to the turbostratic phase, indicating the presence of small, disordered graphitic domains with a locally layered structure [41]. The development of the broad diffraction peak near 43° is a clear indicator of structural evolution driven by pyrolysis temperature. Its absence in the 400 °C sample signifies a highly disordered, amorphous structure. As the temperature increases, this peak becomes progressively more prominent, reaching its maximum intensity at 800 °C. This trend can be explained by a thermally driven reorganization of the carbonaceous matrix. Elevated temperatures promote the growth and ordering of aromatic systems [42]. Furthermore, consistent with the mechanism described by Kennedy et al., the thermal decomposition of less stable C regions during pyrolysis preferentially creates porosity along the incipient graphitic structures [43]. This process effectively removes the amorphous, disordered phase and leaves behind a more stable, organized aromatic network. Therefore, the intensifying peak at 43° directly reflects the formation of a more structurally ordered and stable carbonaceous skeleton as the synthesis temperature increases.

The sharp diffraction peak near 29° corresponds to the mineral calcite (CaCO_3_) [44]. This finding is consistent with XRF analysis of the resulting ash, which showed Ca to be the predominant inorganic component (59.58% ± 1.72). The evolution of this CaCO_3_ peak with temperature is attributed to the thermal crystallization and subsequent transformation of the mineral phase. At 400 °C, the peak is negligible, likely because the Ca species within the carbonaceous matrix did not have sufficient time or thermal energy to fully crystallize into a distinct, detectable CaCO_3_ phase. This is further supported by EDS analysis, where crystalline deposits in the 400 °C sample showed only a minimal Ca content. At higher intermediate temperatures (500–700 °C), the increased thermal energy promotes the crystallization of Ca into well-defined CaCO_3_, resulting in a sharp XRD signal. However, by 800 °C, this peak disappears completely. This final disappearance is consistent with the thermal decomposition of CaCO_3_, which becomes significant above approximately 750 °C, converting CaCO_3_ to CaO [45]. The resulting CaO, being amorphous or highly dispersed within the porous carbonaceous matrix, does not produce a sharp, characteristic XRD peak under these conditions.

### 3.4. Functional Groups Analysis

**Figure 5:**
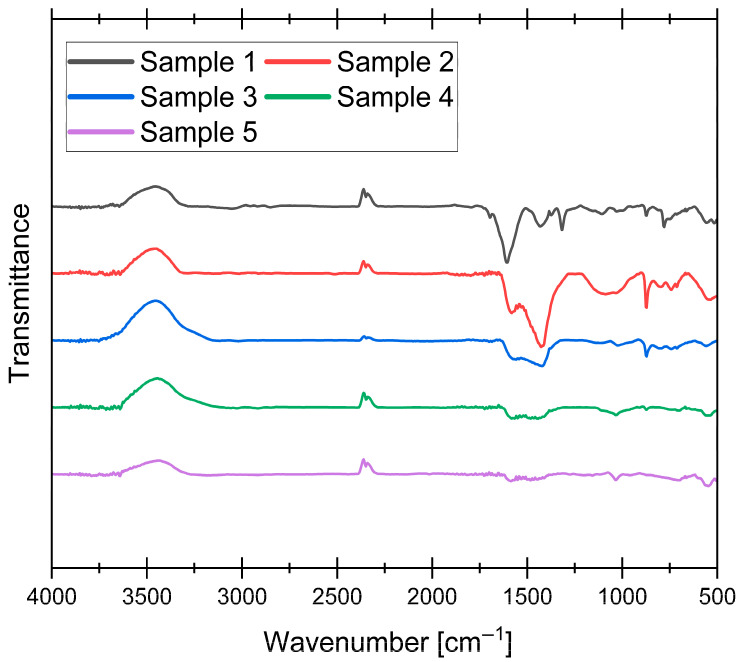
FT-IR spectra of synthesized samples.

FT-IR is an analytical technique widely used for the characterization of materials based on their molecular composition. In the context of AC derived from grape seeds, FT-IR analysis provides valuable insights into the chemical structure and functional groups present in the material, thereby enabling a comprehensive understanding of its properties and potential applications. To determine the impact of synthesis parameters on the mentioned properties of our samples, we analyzed all of them, and the results are shown in Figure 5 (raw spectra without baseline corrections are presented in Supplementary Data Figure S1).

**Figure 6:**
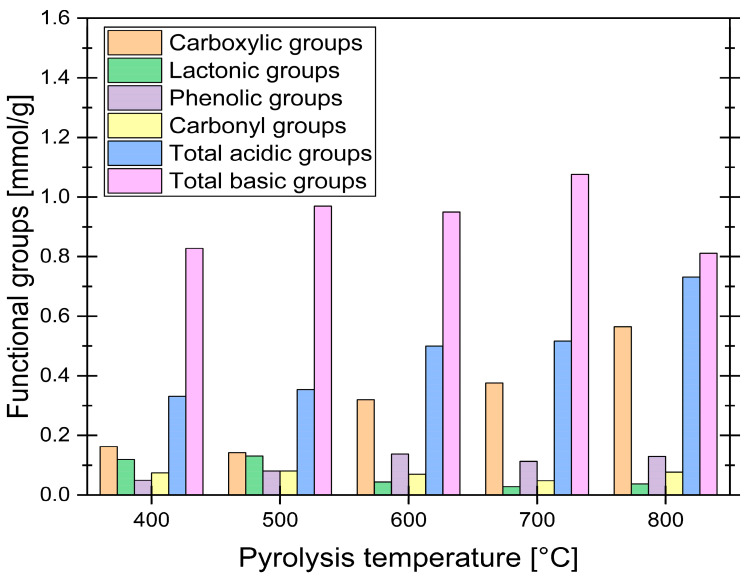
Boehm titration results for synthesized samples.

The FT-IR spectra shown in Figure 6 reveal significant changes in the surface functional groups of the obtained AC samples. A broad feature near 3456 cm^−1^, appearing as an inverted peak in the transmittance spectrum, could be attributed to O-H stretching vibrations from hydroxyl groups on the AC surface, which are common in carbonaceous materials. However, it may also arise from atmospheric water vapor interference, a known artifact in FT-IR measurements. The small inverted double peak near 2350 cm^−1^ arises from CO_2_ and therefore does not provide information about the functional groups of the AC [46]. The region between 1608 and 1573 cm^−1^ corresponds to C=C stretching vibrations in aromatic rings, a characteristic feature of the carbonaceous matrix. This peak is most prominent for sample 1, which showed the lowest degree of graphitization in XRD analysis, suggesting that it most likely corresponds to the disordered, turbostratic phase within the matrix. The shift to lower wavenumbers (except for the 400 °C sample) may reflect changes in the electronic environment of the aromatic system, likely due to the progressive structural reorganization at higher pyrolysis temperatures. Peaks in the range of 1431–1408 cm^−1^ (most prominent in sample 2) are associated with C-H bending vibrations in aliphatic or aromatic structures, with the slight shift toward lower wavenumbers (relative to the 400 °C sample) suggesting minor structural modifications. A distinctive peak at 1315 cm^−1^, observed exclusively in the 400 °C sample, is assigned to C-O stretching vibrations in phenolic, lactonic, or carboxylic groups [47]. Additionally, the peak at 873 cm^−1^ is attributed to out-of-plane C-H bending vibrations in aromatic rings [48].

To further characterize the surface chemistry of the synthesized ACs, Boehm titration was performed. This method quantitatively measures the concentration of acidic and basic functional groups by selectively neutralizing them with bases and acids of varying strengths. The results of this analysis are presented in Figure 6.

The Boehm titration results reveal significant trends in the distribution of functional groups on the surface of ACs derived from grape seeds, influenced by pyrolysis temperature. As the pyrolysis temperature increased from 400 °C to 800 °C, the concentration of carboxylic groups rose substantially, from 0.163 mmol/g to 0.565 mmol/g, while lactonic groups decreased from 0.119 mmol/g to 0.037 mmol/g, suggesting their thermal decomposition. Phenolic groups showed a slight increase, peaking at 600 °C before declining at higher temperatures, while carbonyl groups remained relatively stable. The total acidic groups increased with temperature, driven primarily by the rise in carboxylic groups, while the total basic groups exhibited a slight increase up to 700 °C before decreasing at 800 °C. Research suggests that functional groups undergo decomposition at elevated temperatures [49], but the obtained results indicate that the concentration of some groups, such as carboxylic groups, actually increases with temperature. This could be explained by the decomposition of certain groups leaving active sites on the sample surface [47], which may then react with O_2_ and H_2_O in the air after synthesis, potentially forming new functional groups [50]. This mechanism was directly observed by Menéndez et al., who noted that high-temperature treatment in an inert atmosphere leaves highly reactive, unsaturated carbon atoms at crystallite edges; these sites are very active for subsequent O_2_ adsorption upon air exposure [51]. This makes it challenging to determine which groups are retained from the pyrolysis process and which are newly formed upon exposure to air.

It is worth noting that the Boehm titration results may not always align exactly with FT-IR findings, as literature suggests that such correlations are not always straightforward [52]. Nevertheless, the combined use of these methods provides a more comprehensive understanding of the surface chemistry of AC, which is essential for tailoring its properties for specific applications, such as adsorption or catalysis.

### 3.5. PZC Analysis

**Figure 7:**
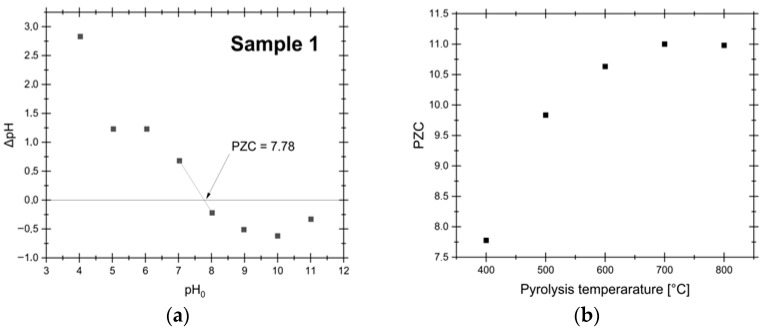
PZC analysis results for samples synthesized at varying temperatures (400–800 °C): (**a**) results for the sample synthesized at 400 °C, shown here as a representative case; (**b**) summary of the results for all temperatures.

The surface chemistry of AC is pivotal in determining its adsorption performance. In addition to functional groups, the PZC is another critical parameter that influences the AC adsorption behavior, particularly in aqueous environments. The PZC, defined as the pH at which the net surface charge is zero, dictates whether the AC surface is positively or negatively charged under specific pH conditions, thereby affecting its affinity for cationic or anionic species. The results for PZC analysis are presented in Figure 7.

The findings suggest that higher pyrolysis temperatures favor the development of a more basic surface character, as indicated by the increase in PZC from 7.78 at 400 °C to 11.00 at 800 °C. This shift enhances the AC ability to adsorb anionic species in acidic solutions, as the positively charged surface attracts negatively charged complexes at pH < PZC. In the Pd-Cl system under highly acidic conditions (pH = 1) with a chloride concentration of 0.1 M, the PdCl_4_^2−^ complex is the only stable species in the system [29]. Since this complex is anionic and the AC surface is positively charged at pH values below the PZC, the adsorption of PdCl_4_^2−^ is expected to occur with high efficiency due to strong electrostatic attraction.

This observed increase in PZC with pyrolysis temperature is consistent with trends reported in the literature, where higher synthesis temperatures often yield ACs with more basic surfaces. Conventionally, this effect has been attributed to changes in surface functional group chemistry [53,54]. However, Boehm titration data from this work revealed a concurrent increase in the concentration of acidic groups—predominantly carboxylic acids—with increasing pyrolysis temperature. Since acidic groups lower PZC, their increase directly opposes the measured rise in PZC, indicating that the evolution of those functional groups is not the driver of the enhanced basicity in this system. Instead, this apparent contradiction points to the influence of inorganic constituents. Elemental analysis showed a significant increase in ash content with temperature, primarily composed of alkaline metals such as Ca. These inorganic salts, remaining within the carbonaceous matrix after pyrolysis, can impart a pronounced alkaline character to the material [55,56], therefore elevating its effective PZC.

### 3.6. Pd(II) Adsorption Test

The adsorption of Pd(II) onto grape-derived AC at pH = 1 was investigated for all samples. Given that leachate solutions from secondary Pd recovery processes are often highly acidic [57,58], it was considered practical to evaluate AC performance under acidic conditions. The experimental data were analyzed using the Freundlich and Temkin isotherms (Equations (4) and (5)). The maximum experimental adsorption capacity (q_e,_ max) was determined as well, as this parameter is commonly used to compare the performance of different adsorbents. The Langmuir isotherm was also investigated, but the obtained results did not fit the model well, suggesting that the adsorption mechanism does not follow the assumptions of monolayer adsorption on a homogeneous surface. Instead, the Freundlich and Temkin models provided a better description of the adsorption process. The compiled data are presented in Table 4, while the exact concentrations of Pd during the adsorption test are compiled in Table S1.

The Freundlich isotherm was found to provide an excellent fit to the experimental data, as evidenced by the high correlation coefficients (R^2^ > 0.91 for all samples and temperatures). The Freundlich adsorption constant K_F_ increased with temperature for all samples, reflecting an improvement in the adsorption capacity of AC for Pd at higher temperatures. For example, for Sample 1, K_F_ increased from 0.0086 at 30 °C to 0.0860 at 50 °C. This trend suggests that the adsorption process is endothermic, meaning it requires heat to proceed, and higher temperatures favor greater adsorption. The Freundlich exponent (*n*) ranged from 0.693 to 1.349 across all samples and temperatures. Values of *n* > 1 indicate favorable adsorption, while *n* < 1 suggests less favorable adsorption or a heterogeneous surface. For most samples, *n* increased with temperature, further supporting the endothermic nature of the adsorption process.

The Temkin isotherm also provided a good fit to the experimental data, with R^2^ values generally above 0.9, except for Sample 1 at 30 °C. This model accounts for the effects of adsorbent–adsorbate interactions and assumes that the heat of adsorption decreases linearly with coverage. The Temkin constant (A_T_) increased with temperature, reflecting an increase in the equilibrium binding constant at higher temperatures. For Sample 1, A_T_ increased from 0.076 L/g at 30 °C to 0.102 L/g at 50 °C. This trend is consistent with the Freundlich results and further confirms the endothermic nature of the adsorption process. The Temkin constant (b_T_) decreased with temperature, indicating that the heat of adsorption decreases as temperature increases. For Sample 1, b_T_ decreased from 0.822 kJ/mol at 30 °C to 0.417 kJ/mol at 50 °C. This suggests that the adsorbent–adsorbate interactions weaken at higher temperatures, which is typical for physical adsorption processes.

The experimental maximum adsorption capacity (q_e, max_) showed a clear increase with temperature for all samples. For example, for Sample 1, q_e, max_ increased from 7.76 mg/g at 30 °C to 16.20 mg/g at 50 °C. For Sample 5, q_e, max_ increased from 4.73 mg/g at 30 °C to 11.45 mg/g at 50 °C. This shows that for the purpose of Pd(II) recovery, pyrolysis at 400 °C is the most optimal (among the tested parameters) to produce AC from grape seeds. Despite possessing the lowest BET surface area, sample 1 exhibited optimal adsorption performance at 400 °C. This indicates that a well-developed pore network is not the primary factor governing adsorption for this specific adsorbate. Sample 1 differs from the others primarily in its lowest PZC, lowest ash content, and lowest degree of graphitization. However, none of these properties adequately explain its significantly greater adsorption capacity. The most plausible explanation is that Sample 1 possesses a distinct profile of basic surface functional groups. This is proposed despite its total quantity of basic groups—as measured by Boehm titration—not being the highest among the samples. At pH = 1, basic functional groups are expected to play a dominant role in the adsorption mechanism [59], suggesting that the specific type or arrangement of these groups, rather than their total concentration, is critical for the observed performance.

To contextualize the performance of our AC, we compared it with data from the literature for Pd(II) adsorption on various ACs in an HCl medium. A summary of the maximum adsorption capacities reported in other studies is presented in Table 5.

The adsorption capacity of our grape seed-derived AC (16.20 mg/g at 50 °C) compares reasonably well with commercial and literature-reported ACs, despite requiring no chemical or physical activation. While lower than some high-performance steam ACs like coconut shell (67.0 mg/g) and wood charcoal (111.3 mg/g), our material matches or exceeds other biomass-derived alternatives (15.6 mg/g for cherry seeds) and even outperforms certain steam-activated materials (27.0 mg/g for peat). This demonstrates that our simplified one-step pyrolysis can produce an effective adsorbent that approaches the performance of more energy-intensive commercial materials, making it a promising sustainable alternative for Pd(II) recovery applications where moderate capacity is acceptable. The relatively small performance gap is particularly notable given our method’s avoidance of activation steps and use of waste biomass as feedstock.

### 3.7. LCA Analysis

The LCA analysis was employed to evaluate the environmental impacts associated with the system under study. Since our study did not include analysis of gas composition during pyrolysis, we based these calculations on the comprehensive work of Veses et al. [63], who thoroughly characterized the grape seed pyrolysis process. Given that the AC synthesized at 400 °C (Sample 1) demonstrated the highest Pd(II) adsorption capacity, it was selected as the representative case for this environmental assessment. To contextualize the sustainability of the produced AC, its environmental performance was compared to that from the database mentioned in the Section 2. The analysis does not take transportation issues into account in its assumptions.

The functional unit of calculation is one kilogram of AC produced. The analysis does not include transportation data. The model is based on a dataset of electricity corresponding to the Polish energy mix (30% renewable energy sources and 70% coal-based electricity), while the amount of energy used to produce AC, due to the small amount of the sample in relation to the total capacity of the furnace, and because a laboratory-grade furnace was used for the experiment, was calculated based on the amount of energy absorbed by the sample during the pyrolysis process. The system boundaries of the analysis are a cradle-to-gate approach. The inventory data for comparative analysis are presented in Table S2.

These experimental data served as the fundamental basis for generating the environmental impact results through LCA modeling. The comparative LCA uses a cut-off system model for granular AC production from hard coal (Ecoinvent dataset: “Activated carbon, granular {RoW}|activated carbon production, granular from hard coal|Cut-off, S”). The process requires 3 kg of hard coal to produce 1 kg of AC, with emissions modeled using hard coal combustion (1–10 MW industrial furnace, 80% efficiency, 28.9 MJ/kg heating value). Granular AC production from hard coal, typical for Central Europe, involves carbonization at temperatures over 700 °C followed by activation via vaporization at 800–1000 °C. The activation stage, critical for developing porosity, consumes 1.6 kWh of electricity and 0.33 m^3^ of natural gas to heat 12 kg of water per 3 kg of coal processed. Emissions from carbonizing the excess 2 kg of hard coal were derived from the dataset “heat production, at hard coal industrial furnace 1–10 MW, GLO 1992,” which assumes a furnace lifetime of 20 years (5000 full-load hours/year) burning egg coal with 28.9 MJ/kg heating value. Data sources include Bayer et al. on granular AC refill strategies [64] and Muñoz et al. on LCA comparisons with adsorption processes [65].

**Figure 8:**
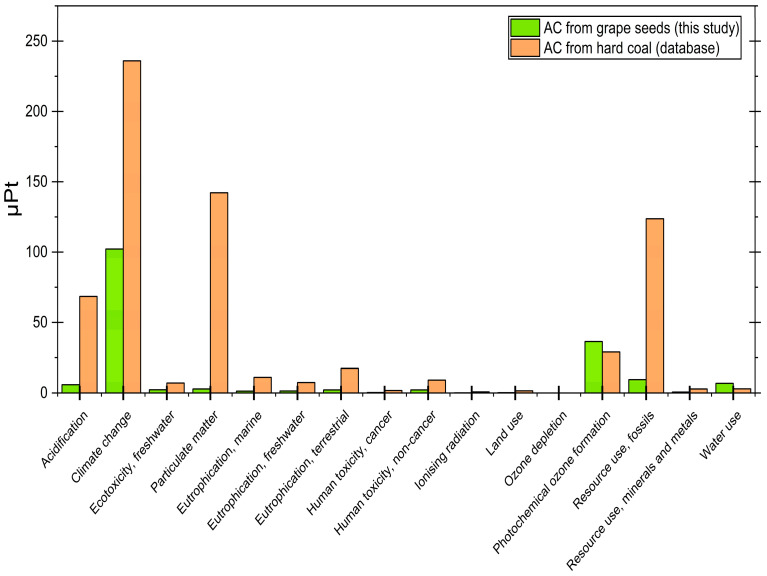
LCA result comparison.

The results are presented in two forms: Table S3 displays the original impact category values, while Table S4 provides the normalized comparison in ecopoints (µPt), where weighting factors are applied to enable cross-category evaluation. For additional clarity, the weighted analysis using ecopoints is also presented graphically in Figure 8.

The grape seed-derived AC demonstrates superior environmental performance across nearly all impact categories when compared to the commercial reference material. Most strikingly, the total environmental impact of our bio-based AC (173.87 µPt) is 74% lower than that of the conventional material (661.11 µPt). This advantage is particularly pronounced in several key categories: acidification potential is dramatically reduced (5.90 µPt vs. 68.48 µPt, a 91% improvement), climate change impact is less than half (102.14 µPt vs. 235.94 µPt), and particulate matter formation shows a remarkable 98% reduction (2.82 µPt vs. 142.14 µPt). The fossil resource use is just 8% of the commercial equivalent (9.41 µPt vs. 123.70 µPt), highlighting significant energy efficiency advantages in the production process.

Beyond these core impact categories, additional benefits are observed across toxicity and eutrophication indicators. For instance, human toxicity (cancer and non-cancer effects) is consistently reduced (0.30 µPt vs. 1.75 µPt and 2.15 µPt vs. 9.14 µPt, respectively). Similar reductions occur for eutrophication across freshwater (1.39 µPt vs. 7.35 µPt), marine (1.27 µPt vs. 11.14 µPt), and terrestrial ecosystems (2.15 µPt vs. 17.34 µPt). Even in categories such as ecotoxicity (2.23 µPt vs. 7.12 µPt), ionizing radiation (0.09 µPt vs. 0.74 µPt), and land use (0.18 µPt vs. 1.51 µPt), the grape seed-derived material consistently outperforms the hard-coal reference.

Two exceptions to this positive trend require specific consideration. Photochemical ozone formation shows a modest 26% increase (36.37 µPt vs. 28.97 µPt), suggesting potential opportunities for process optimization in emission control during manufacturing. However, it is worth mentioning the water consumption of both processes, where in the case of in-house production, a difference of approximately 56% (6.85 µPt vs. 2.98 µPt) is observed. This area indicates the possibility of improvement, e.g., by introducing a system for recovering water from waste gases during grain roasting, or a steam heat recovery system that would recover not only water but also valuable energy resources, depending on the scale of the project.

A key advantage of the analyzed process is its reliance on biogenic waste material as feedstock, which substantially reduces its environmental footprint. The modeled calculations confirm this hypothesis. Additionally, the process is relatively simple and does not require the use of primary raw materials extracted from mining operations, as is the case with the database reference process. In a theoretical industrial or pilot-scale implementation, the emissions from the analyzed process could be further reduced by utilizing an alternative heat source, such as waste heat from another industrial process. Alternatively, if an electric furnace is used, the environmental impact could be minimized by sourcing electricity exclusively from renewable energy sources.

The overall results clearly demonstrate that grape seed-derived AC offers compelling environmental advantages for applications where climate change mitigation, acidification reduction, and resource efficiency are prioritized. While the water use impact presents a notable challenge, strategic improvements in water recycling and process efficiency could further enhance the sustainability profile. These findings position bio-based AC as a promising sustainable alternative, particularly in regions where water scarcity is less critical than atmospheric emissions and resource conservation.

Although direct LCA studies on grape seed-derived AC remain limited, analyses of other biomass-derived ACs—such as those from coconut shells and olive pomace—provide valuable insights. For instance, an LCA of olive pomace-based AC revealed that the phosphoric acid (H_3_PO_4_) impregnation stage contributed significantly to environmental impacts, accounting for 62% of acidification potential and 96% of eutrophication potential, primarily due to electricity and chemical consumption [66]. Similarly, coconut shell-based AC production demonstrated that renewable energy integration could reduce greenhouse gas emissions by up to 80% [67]. These parallels suggest that optimizing energy sources and chemical use in grape seed activation could further improve its environmental performance.

## 4. Conclusions

This study demonstrates that AC suitable for Pd(II) recovery can be produced from grape seed waste via a single-step pyrolysis process without chemical or physical activation. Among the investigated synthesis temperatures (400–800 °C), the sample obtained at 400 °C exhibited the highest Pd(II) adsorption capacity (16.20 mg g^−1^ at 50 °C in 0.1 M HCl), despite having the lowest BET surface area, confirming that adsorption of Pd–chloro complexes under strongly acidic conditions is governed primarily by surface chemistry rather than textural properties. Comprehensive characterization showed that increasing pyrolysis temperature resulted in higher ash content, greater structural ordering, and increased PZC values due to the accumulation of alkaline inorganic species such as Ca; however, these changes did not lead to improved Pd(II) uptake. From a sustainability perspective, LCA revealed clear environmental advantages of the proposed approach, with grape seed-derived AC exhibiting a 74% lower total environmental impact and a 92% reduction in acidification potential compared to conventional coal-based AC, mainly due to the elimination of activation steps, reduced energy demand, and the use of biogenic waste as feedstock. Overall, the results demonstrate that low-temperature pyrolysis of winemaking waste can yield an effective and environmentally favorable adsorbent for Pd(II) recovery from acidic solutions, highlighting the potential of biomass-derived AC produced via a single-step pyrolysis route as a viable alternative to conventional AC production practices.

## Figures and Tables

**Table 1:** Ash content results.

Sample Number	AC Mass [g]	Ash Mass [g]	Ash Content [%]
1	1.002	0.047	4.66
2	0.801	0.046	5.80
3	0.709	0.038	5.34
4	0.681	0.037	5.39
5	0.711	0.040	5.68

**Table 2:** EDXRF results for the obtained ashes.

Element [%]	Sample Number					Mean ± SD
	1	2	3	4	5	
Ca	57.60	60.90	57.90	59.40	62.10	59.58 ± 1.72
P	23.90	20.10	23.80	21.70	20.60	22.02 ± 1.77
K	8.90	9.60	8.40	9.00	8.00	8.78 ± 0.61
Mg	5.40	5.00	6.10	5.60	5.30	5.48 ± 0.41
S	2.70	2.80	2.20	2.70	2.40	2.56 ± 0.25
Fe	1.10	1.20	1.20	1.10	1.20	1.16 ± 0.05
Cu	0.13	0.13	0.15	0.13	0.13	0.13 ± 0.01
Zn	0.10	0.10	0.10	0.20	0.10	0.12 ± 0.04
Mn	0.12	0.13	0.11	0.12	0.12	0.12 ± 0.01
Cr	0.03	0.02	0.02	0.02	0.02	0.022 ± 0.004
Ti	0.02	0.02	0.02	0.03	0.03	0.024 ± 0.005

**Table 3:** EDS analysis results.

Points	Elemental Content (at.%)							
	C	N	O	Mg	Si	P	K	Ca
001	66.31	19.24	10.26	0.41	0.07	1.26	0.72	1.73
002	84.31	6.31	6.00	0.60	0.18	1.75	0.46	0.39
003	56.05	15.60	26.88	0.11	0.06	0.24	0.08	0.98
004	54.76	0.00	41.05	0.00	0.10	0.09	0.05	3.95
005	58.08	6.12	31.36	0.03	0.05	0.09	0.05	4.22
006	79.14	4.64	14.36	0.16	0.00	0.26	0.23	1.21
007	63.54	19.56	12.34	0.11	0.73	0.47	2.82	0.43
008	81.46	4.49	10.86	0.53	0.36	1.53	0.44	0.33
009	83.65	4.78	11.19	0.00	0.11	0.06	0.18	0.03
010	66.15	3.72	17.91	0.43	1.10	1.38	2.91	6.40
011	38.78	0.00	13.71	0.09	0.25	1.13	0.57	45.47
012	64.83	0.00	21.50	0.13	0.09	1.11	7.80	4.54
013	63.26	0.00	23.82	0.62	0.16	4.47	0.36	7.31
014	79.71	0.00	15.68	0.92	0.21	1.50	0.42	1.56
015	56.03	0.00	26.19	0.55	0.18	6.75	0.31	9.99

**Table 4:** Pd(II) adsorption results—Freundlich and Temkin isotherms parameters and experimental maximum adsorption capacities.

Temperature [°C]	Freundlich Isotherm			Temkin Isotherm			Experimental Maximum Adsorption Capacity
	K_F_ [(mg/g) (L/mg)1/n]	n	R^2^	A_T_ [L/g]	b_T_ [kJ/mol]	R^2^	q_e_, Max [mg/g]
Sample 1							
30	0.0086	0.693	0.999	0.0761	0.822	0.873	7.76
40	0.0468	0.842	0.986	0.0932	0.591	0.937	11.27
50	0.0860	0.863	0.995	0.1019	0.417	0.954	16.20
Sample 2							
30	0.0277	0.891	0.955	0.0926	1.183	0.977	5.29
40	0.0729	1.009	0.975	0.1050	0.920	0.989	7.52
50	0.1061	1.046	0.932	0.1180	0.832	0.998	8.49
Sample 3							
30	0.0517	1.034	0.957	0.1064	1.378	0.978	4.69
40	0.0862	1.052	0.994	0.1011	0.875	0.916	8.47
50	0.1819	1.139	1.000	0.1171	0.653	0.937	11.92
Sample 4							
30	0.0698	1.162	0.971	0.1128	1.704	0.968	3.98
40	0.0876	1.065	0.993	0.1069	0.984	0.940	7.26
50	0.1754	1.141	0.986	0.1261	0.674	0.932	10.49
Sample 5							
30	0.1344	1.349	0.997	0.1288	1.590	0.936	4.73
40	0.1525	1.199	0.987	0.1224	0.962	0.942	7.79
50	0.2516	1.229	0.996	0.1317	0.684	0.931	11.45

**Table 5:** Literature summary of maximum Pd(II) adsorption capacities on ACs.

q_e_, Max [mg/g]	AC Origin	AC Activation	Medium	AC Dose [g/L]	C_0_ of Pd(II) [mg/L]	Temperature [°C]	Reference
16.2	Grape seeds	None	0.1 M HCl	1.67	106.42	50	This study
15.6	Cherry seeds	None	0.1 M HCl	1.67	212.84	50	[15]
67.0	Coconut shell	Steam	0.1 M HCl	1.67	239.45	50	[29]
27.0	Peat	Steam	2 M HCl	7.50	225.00	25	[60]
51.6	Bituminous coal	Steam	0.1 M HCl	0.61	50.00	20	[57]
111.3	Wood charcoal	Steam	0.1 M HCl	1.60	266.05	21	[61]
81.1	Coconut shell	Not specified	0.1 M HCl	1.66	42.57	21	[62]
41.39	Coconut shell	Steam	0.1 M HCl	0.70	50.00	20	[57]

## Data Availability

The original contributions presented in the study are included in the article/Supplementary Material, further inquiries can be directed to the corresponding author.

## Supplementary Materials

The following supporting information can be downloaded at: https://www.mdpi.com/article/10.3390/ma19010107/s1, Figure S1. Raw FT-IR spectra for AC samples. Table S1. Pd concentrations during the adsorption tests. Table S2. Inventory data for comparative analysis. Table S3. Characterized results of LCA analysis of AC from grape seeds and commercial AC (from database). Table S4. Weighted results of LCA analysis of AC from grape seeds and commercial AC (from database).
