# Supplementary material for: Industrial Winemaking Waste to Sustainable Palladium(II) Recovery: A Green One-Step Synthesis of Activated Carbon from Grape Seeds
Source: Materials (Basel). 2025 Dec 28;19(1):107. doi: 10.3390/ma19010107 (PMC12786859; doi:10.3390/ma19010107)
Supplement: Supplementary file 1 [file materials-19-00107-s001.zip › materials-4041047-supplementary.pdf]

Supplementary Data

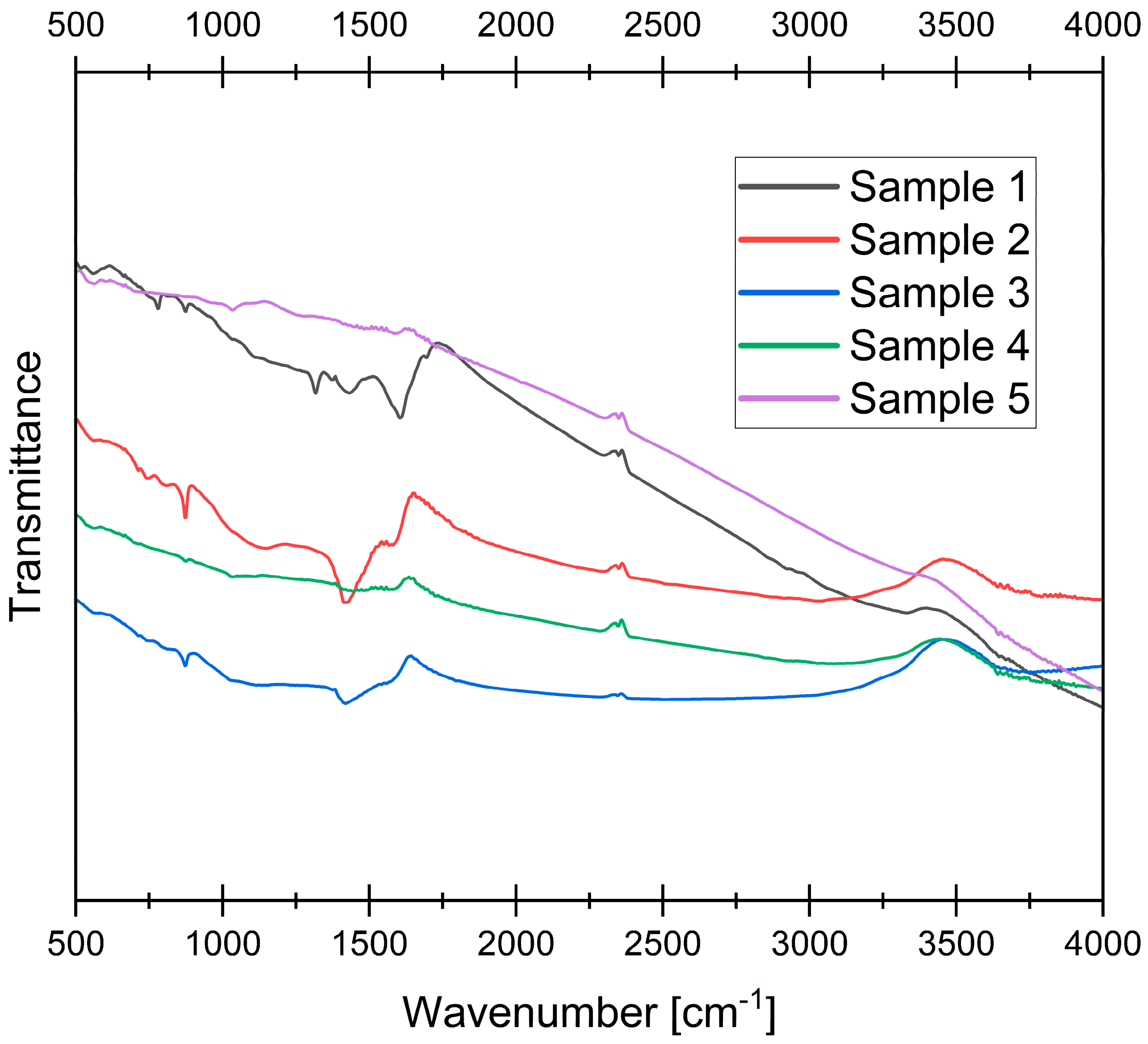

Figure S1. Raw FT-IR spectra for AC samples.

Table S1. Pd concentrations during the adsorption tests.

Sample number				
1	2	3	4	5
Initial Pd concentrations [mg/L]				

11.83506	11.93273	11.89687	11.93265	12.00007
18.38186	18.55011	18.56979	18.58436	18.6124
62.20925	62.17272	62.83455	62.15629	62.31468
125.1312	135.8677	137.7246	123.9572	124.5207
Pd concentrations after the adsorption at 30°C [mg/L]				
11.38387	11.18183	11.03904	10.90319	10.64987
17.42673	17.63478	17.34037	17.46975	16.85794
57.34849	56.16627	56.86549	57.75595	57.93213
112.1753	127.0283	129.89	117.3121	116.6282
Pd concentrations after the adsorption at 40°C [mg/L]				
10.73064	10.83584	10.63944	10.69516	10.38666
15.862	16.34157	16.28629	16.32974	15.69681
53.13458	54.53434	56.4701	55.86429	55.35668
106.323	123.3015	123.5685	111.8397	111.511
Pd concentrations after the adsorption at 50°C [mg/L]				
9.936505	10.65708	9.687717	9.563656	9.268675
14.89369	15.48013	15.22489	15.7763	15.04603
48.1409	52.06732	52.91112	52.27365	52.05107
98.08347	121.6727	117.8084	106.4605	105.397

Table S2. Inventory data for comparative analysis.

Name of input/output	Input/output	Type of input/output	Quantity used/produced	Unit
Grape seeds	Input	Material	17.16	g
Electricity	Input	Material	0.00383	kWh
Hydrogen	Output	Product	0.594	g
Carbon monoxide	Output	Emmision to air	0.609	g
Carbon dioxide	Output	Emmision to air	1.328	g
Methane	Output	Emmision to air	0.666	g
Ethane	Output	Emmision to air	0.362	g

Propane	Output	Emmision to air	0.145	g
Butane	Output	Emmision to air	0.099	g
Organic waste	Output	To treatment facility	6.971	g

Table S3. Characterized results of LCA analysis of AC from grape seeds and commercial AC (from database).

**Impact category**	**Unit**	**Activated carbon from grape seeds**	**Activated carbon (from database)**
Acidification	mol H^+ eq	0.005284	0.061381
Climate change	kg CO_2 eq	3.663129	8.461976
Climate change - Biogenic	kg CO_2 eq	2.985410	0.002668
Climate change - Fossil	kg CO_2 eq	0.678	8.456079
Climate change - Land use and LU change	kg CO_2 eq	0.000187	0.003228
Ecotoxicity, freshwater - part 1	CTUe	2.18	18.42629
Ecotoxicity, freshwater - part 2	CTUe	4.40801	2.594089
Ecotoxicity, freshwater - inorganics	CTUe	6.43	20.62532
Ecotoxicity, freshwater - organics - p.1	CTUe	0.0254	0.124676
Ecotoxicity, freshwater - organics - p.2	CTUe	0.132242	0.270385
Particulate matter	disease inc.	1.87×10^{-8}	9.44×10^{-7}
Eutrophication, marine	kg N eq	0.000839	0.007356
Eutrophication, freshwater	kg P eq	7.96×10^{-5}	4.22×10^{-4}
Eutrophication, terrestrial	mol N eq	0.010234	0.082616
Human toxicity, cancer	CTUh	2.44×10^{-10}	1.42×10^{-9}
Human toxicity, cancer - inorganics	CTUh	1.56×10^{-10}	9.78×10^{-10}
Human toxicity, cancer - organics	CTUh	8.73×10^{-11}	4.40×10^{-10}
Human toxicity, non-cancer	CTUh	1.50×10^{-8}	6.39×10^{-8}
Human toxicity, non-cancer - inorganics	CTUh	9.43×10^{-9}	6.22×10^{-8}

Human toxicity, non-cancer - organics	CTUh	5.61×10^{-9}	1.76×10^{-9}
Ionising radiation	kBq U-235 eq	0.007649	0.062401
Land use	Pt	1.840966	15.63204
Ozone depletion	kg CFC11 eq	1.02×10^{-8}	2.20×10^{-8}
Photochemical ozone formation	kg NMVOC eq	0.031090	0.024766
Resource use, fossils	MJ	7.350701	96.64319
Resource use, minerals and metals	kg Sb eq	5.22×10^{-7}	2.34×10^{-6}
Water use	m^3 depriv.	0.923658	0.402257

Table S4. Weighted results of LCA analysis of AC from grape seeds and commercial AC (from database).

Impact Category	Unit	Activated Carbon from Grape Seeds	Activated Carbon (from database)
**Total**	µPt	174.00	661.00
**Acidification**	µPt	5.90	68.50
**Climate Change**	µPt	102.00	236.00
**Ecotoxicity, Freshwater**	µPt	2.23	7.12
**Particulate Matter**	µPt	2.82	142.00
**Eutrophication, Marine**	µPt	1.27	11.10
**Eutrophication, Freshwater**	µPt	1.39	7.35
**Eutrophication, Terrestrial**	µPt	2.15	17.30
**Human Toxicity, Cancer**	µPt	0.30	1.75
**Human Toxicity, Non-cancer**	µPt	2.15	9.14
**Ionising Radiation**	µPt	0.090805	0.74
**Land Use**	µPt	0.178	1.51
**Ozone Depletion**	µPt	0.0123	0.03

**Photochemical Ozone Formation**	μPt	36.4	29.00
**Resource Use, Fossils**	μPt	9.41	124.00
**Resource Use, Minerals/Metals**	μPt	0.619	2.78
**Water Use**	μPt	6.85	2.98
